# Adherence to and clinical utility of “quality indicators” for *Staphylococcus aureus* bacteremia: a retrospective, multicenter study

**DOI:** 10.1007/s15010-024-02284-z

**Published:** 2024-05-10

**Authors:** Shinnosuke Fukushima, Hideharu Hagiya, Naoki Kuninaga, Yuto Haruki, Haruto Yamada, Yoshitaka Iwamoto, Masayo Yoshida, Kota Sato, Yoshihisa Hanayama, Shuichi Tanaka, Tomoko Miyoshi, Yuki Otsuka, Keigo Ueda, Fumio Otsuka

**Affiliations:** 1 Department of General Medicine, Okayama University Graduate School of Medicine, Dentistry and Pharmaceutical Sciences, Okayama, Japan; 2 Department of Bacteriology, Okayama University Graduate School of Medicine, Dentistry and Pharmaceutical Sciences, Okayama, Japan; 3 Department of Infectious Diseases, Okayama University Hospital, 2-5-1 Shikata-Cho, Kitaku, Okayama, 700-8558 Japan; 4 Department of General Medicine, Kurashiki Central Hospital, Okayama, Japan; 5 Department of Pharmacy, Tsuyama Chuo Hospital, Okayama, Japan; 6 Department of General Medicine, Okayama City Hospital, Okayama, Japan; 7 Department of General Medicine, Okayama Medical Center, Okayama, Japan; 8 Department of General Medicine, Okayama Kyoritsu Hospital, Okayama, Japan; 9 Department of Neurology, Brain Attack Center Ota Memorial Hospital, Fukuyama, Japan

**Keywords:** Bloodstream infection, *Staphylococcus aureus* bacteremia, Prognosis, Quality indicators, Medical safety

## Abstract

**Background:**

We aimed to improve the prognosis, treatment, and management of *Staphylococcus aureus* bacteremia (SAB) by evaluating the association between adherence to quality indicators (QIs) and clinical outcomes in patients with their clinical outcomes.

**Methods:**

We retrospectively collected clinical and microbiological data on hospitalized patients with SAB from 14 hospitals (three with >  600, two with 401–600, five with 201–400, and four with ≤ 200 beds) in Japan from January to December 2022. The SAB management quality was evaluated using the SAB-QI score (ranging from 0 to 13 points), which consists of 13 QIs (grouped into five categories) based on previous literature.

**Results:**

Of the 4,448 positive blood culture episodes, 289 patients with SAB (6.5%) were enrolled. The SAB-QI scores ranged from 3 to 13, with a median score of 9 points. The SAB-QI score was highest in middle-sized hospitals with 401–600 beds. Adherence to each of the four QI categories (blood culture, echocardiography, source control, and antibiotic treatment) was significantly higher in survived cases than in fatal cases. Kaplan–Meier curves with log-rank tests demonstrated that higher adherence to SAB-QIs indicated a better prognosis. Logistic regression analysis revealed that age, methicillin resistance, multiple comorbidities (≥ 2), and low SAB-QI score were significantly associated with 30-day mortality in patients with SAB.

**Conclusions:**

Our study highlights that greater adherence to the SAB-QIs correlates with improved patient outcomes. Management of patients with SAB should follow these recommended indicators to maintain the quality of care, especially for patients with poor prognosticators.

**Supplementary Information:**

The online version contains supplementary material available at 10.1007/s15010-024-02284-z.

## Background

*Staphylococcus aureu*s is a common human pathogen that potentially causes fatal diseases [1, 2], including bacteremia (*S. aureu*s bacteremia, SAB). Patients with SAB frequently experience systemic complications, including abscess formation primarily in the bones, joints, and muscles, along with intravascular infections such as infective endocarditis (IE). Thus, multidisciplinary management, including appropriate drainage/surgery and antibiotic treatment, is essential for patients with SAB [2–5]. Patients with persistent or complicated SAB, in particular, are at greater risk of disseminated infections and higher mortality [6, 7], and sufficient therapeutic approaches are important to prevent recurrence and improve the prognosis of such patients [8, 9]. However, the management of patients with SAB varies among clinicians and hospitals because of the complexity and diversity of the disease, potentially leading to significant gaps in quality of care.

Quality Indicators (QIs) have recently been introduced into clinical practice, surveillance, and evaluation in healthcare institutions to ensure the quality of medical care for various diseases [10–12]. The clinical utility of QIs in managing patients with SAB has recently been reported [13, 14]. Previous studies have shown that interventions, including follow-up blood cultures, early source control, early intravenous cefazolin or anti-penicillin (nafcillin, oxacillin, or cloxacillin) administration for methicillin-susceptible isolates, and appropriate therapy duration, are associated with a decrease in 14- and 30-day mortality [4, 13]. In a single-facility study in Japan, adherence to five QIs for the management of patients with SAB was retrospectively investigated over a 9-year period, including (1) follow-up blood cultures, (2) early source control when applicable, (3) echocardiography, (4) early use of appropriate antibiotics, and (5) appropriate duration of therapy [14]. The mean QI points increased from 3.4 to 4.1 as a result of intervention by infectious disease physicians, leading to a decrease in 30-day mortality from 10.0% to 3.4%.

Recently, international multidisciplinary experts developed “25 QIs for SAB management” to facilitate good care for patients with SAB [10]. Based on the RAND-modified Delphi procedure, potential indicators were extracted from published literature and determined after two rounds of face-to-face expert meetings. However, the clinical evaluation of the updated QIs for SAB management is yet to be performed. Therefore, this study aimed to investigate the association between adherence to the QIs in SAB management and patient prognosis in our clinical setting.

## Methods

### Study design and settings

We retrospectively collected the clinical and microbiological data of patients with SAB from the medical records of 14 hospitals located in Okayama, Hiroshima, and Kagawa prefectures in Japan between January and December 2022. Ethical approval was obtained from the Institutional Review Board of Okayama University Hospital (No. 2302-027). The requirement for informed consent was waived because this was a retrospective analysis of routinely collected and fully anonymized data.

### Inclusion and exclusion criteria

A clinical case of SAB was defined as the isolation of *S. aureu*s from at least one set of blood cultures collected during the study period. Episodes with an interval of 3 months or more were defined as different incidences of SAB [14]. Nosocomial infections were defined as those occurring > 72 h after hospital admission. The exclusion criteria were as follows: (1) patients aged < 18 years; (2) patients not hospitalized for at least 3 days after diagnosis of SAB; (3) patients who died before starting antibiotic administration; (4) patients discharged or transferred from other facilities within 3 days of diagnosis of SAB; (5) those lost to follow-up 14 days after diagnosis of SAB; (6) clinical diagnosis of an SAB episode as contamination; and (7) an episode within an interval of less than 3 months in the same patient.

### Definitions and study protocol

The 25 established QIs for the management of patients with SAB were grouped into five categories: two for *Blood cultures*, six for *Echocardiography*, four for *Non-antibiotic therapeutic interventions*, 11 for *Antibiotic treatment*, and two for *Other management aspects* [10]. In the original literature, several QIs almost overlapped between the uncomplicated and complicated SAB, contributing to increased complexity. To simplify the evaluation, we combined them into 13 SAB-QIs (QIs for the management of SAB) by integrating several indicators of renal function adjustments and source controls without significantly changing their content. The selected indicators are shown in Table 1 as follows: 1–1) follow-up blood cultures, 1–2) confirmation of negative blood culture, 2–1) transthoracic echocardiography (TTE), 2–2) transesophageal echocardiography (TEE), 3) source control, 4–1) intravenous treatment as initial treatment, 4–2) appropriate antibiotics, 4–3) appropriate timing to start antibiotics, 4–4) duration of antibiotics, 4–5) dose adjustment according renal function, 4–6) administration route, 5–1) consultation to infectious disease specialist, and 5–2) description in the medical discharge summary about SAB [13–19]. Each SAB-QI was given 1 point, and we evaluated adherence to SAB-QIs in each enrolled case by calculating the points (range, 0–13 points). We defined the total points for adherence to the QIs as the SAB-QI score.

**Table 1:** Breakdown list of SAB-QIs (Quality Indicators [QIs] for the management of *Staphylococcus aureus* bacteremia [SAB])

QI categories
1) Blood cultures
QI 1–1. Follow-up blood cultures after initiation of antimicrobial therapy
QI 1–2. Collection of repeat blood cultures should be performed until first negative blood culture
2) Echocardiography
QI 2–1. Transthoracic echocardiography should be performed in patients with diagnosed complicated SAB
QI 2–2. Transesophageal echocardiography should be performed in patients with diagnosed complicated SAB
3) Source control
QI 3. After detection of SAB, eradicable focus should be removed
4) Antibiotic treatment
QI 4–1. Initial antibiotic therapy should be administered intravenously in patients with SAB
QI 4–2. Initial therapy should be intravenous cefazolin for MSSA or anti-MRSA drug for MRSA in patients with SAB
QI 4–3. Antibiotic therapy should be initiated within 24 h after first positive blood culture
QI 4–4. Appropriate duration of intravenous antibiotic treatment should be at least 14 days for uncomplicated SAB, or at least 28 days for complicated SAB
QI 4–5. Antibiotic treatment therapy for patients with SAB should be adjusted according to renal function, and vancomycin monitoring should be performed
QI 4–6. Intravenous-to-oral switch should not be performed in < 72 h
5) Other management
QI 5–1. Infectious disease specialist consultation should be performed in patients with SAB
QI 5–2. SAB should be documented in the medical discharge summary

*SAB* *Staphylococcus aureus* bacteremia, *QI* Quality Indicator, *MSSA* methicillin-susceptible *S. aureu*s, *MRSA* methicillin-resistant *S. aureu*s

Uncomplicated SAB was defined as follows: exclusion of endocarditis and other metastatic sites of infection, absence of implanted prostheses, clearance of bacteremia within 4 days in patients with repeated blood cultures, and afebrile status within 72 h after the initiation of effective therapy [10]. Complicated SAB was defined as a case not meeting the criteria for uncomplicated bacteremia and a case without follow-up blood culture [10]. Cases of complicated SAB were further categorized as either confirmed or potentially complicated SAB, in which cases follow-up blood cultures were not obtained. In uncomplicated SAB, echocardiography was not required, meeting the QIs for echocardiography regardless of whether it was performed. The infectious foci were divided into eradicable and ineradicable types. Eradicable foci included surgically removable infections, drainable abscesses, and indwelling foreign bodies such as intravenous catheters and skin abscesses. Ineradicable foci included unknown primary sites, pneumonia, osteomyelitis, or arthritis [20]. The ineradicable foci of SAB were not subjected to QI as 3) source controls. For the treatment of methicillin-susceptible *S. aureu*s (MSSA) infection, cefazolin has been identified as an effective antibiotic, along with anti-staphylococcal penicillins such as nafcillin, oxacillin, and cloxacillin, which are unavailable in Japan [21]. Appropriate antibiotics for methicillin-resistant *S. aureu*s (MRSA) include vancomycin, teicoplanin, and daptomycin. In cases of foreign body-associated infections, such as prosthetic valve endocarditis and prosthetic joint infection, combination therapy with other antimicrobial agents such as rifampicin and gentamicin may be preferred. However, owing to the complexity of data collection, the concurrent use of other agents was not considered in the evaluation. The duration of antibiotic treatment was calculated from the date on which an appropriate antibiotic was initiated after submission of the blood culture. If the patient was transferred to another hospital 3 days after the diagnosis of SAB, the treatment period before transfer was evaluated for QIs. Based on previous studies, we investigated several comorbidities associated with SAB mortality, including diabetes mellitus, immunosuppression (including qualitative deficiency of phagocytic cells, complement, or humoral or cell-mediated immunity), liver cirrhosis, heart failure, malignancy, and chronic renal failure requiring hemodialysis [1, 2, 22]. All patients were monitored until hospital discharge or death. Outcomes 30 days after the diagnosis of SAB were assessed, including outpatient follow-ups and reports from the hospitals to which the patients were transferred. In the analysis, SAB episodes were stratified by the SAB-QI score and categorized into four SAB-QI groups as follows: lowest with 0–6 QIs, lower with 7–8 QIs, higher with 9–10 QIs, and highest with 11–13 QIs.

### Outcome measures and statistical analysis

The primary outcome was the association between 30-day mortality in patients with SAB as defined in previous SAB studies [4, 13, 14] and the SAB-QI score. The secondary outcome was the clinical background of the patients with SAB and the relationship between the SAB-QI score and hospital size, classified by the number of beds (≤ 200, 201–400, 401–600, and > 600 beds).

Continuous variables are described as medians and interquartile ranges (IQRs) and assessed using Kruskal–Wallis or Mann–Whitney U tests. Categorical variables are reported as numbers and percentages and were assessed using the Chi-square or the Fisher's exact test. Comparisons between three or more groups were performed using the Steel–Dwass multiple comparison test. Survival data were analyzed using Kaplan–Meier plots and compared using log-rank tests. We performed logistic regression analysis to identify the factors associated with patient prognosis by incorporating potential variables, including age, site of disease onset, methicillin resistance, complexity of cases, the presence of multiple comorbidities, and SAB-QI score. The data were analyzed using EZR software, a graphic user interface for the R 3.5.2 software (The R Foundation for Statistical Computing, Vienna, Austria) [23]. All reported *p* values < 0.05 were considered statistically significant.

## Results

### Backgrounds

**Fig. 1:**
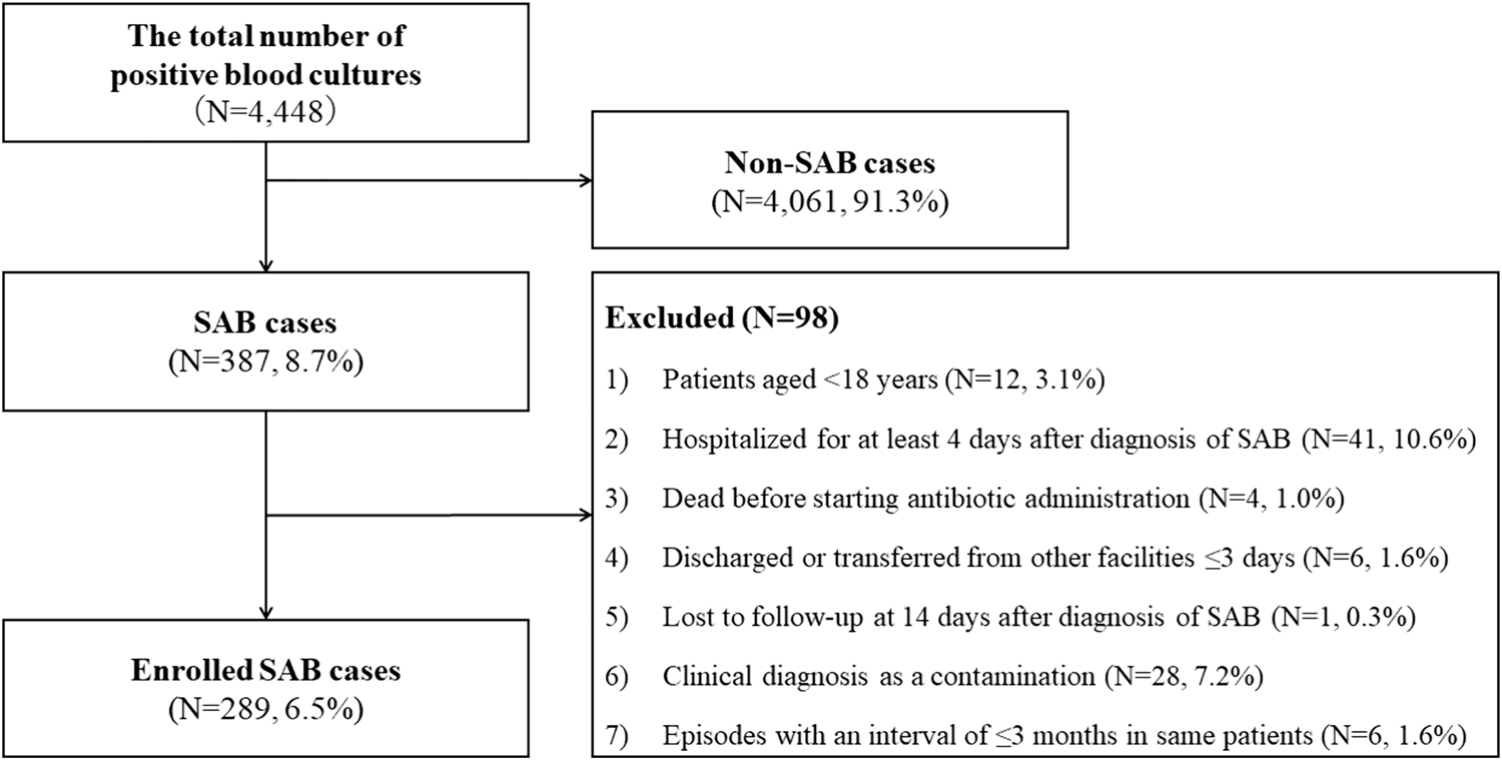
Study enrolment flow. SAB: *Staphylococcus aureus* bacteremia

During the study period, the total number of positive blood cultures was 4,448: 387 cases of SAB (8.7%) and 4,061 of non-SAB bacteremia (91.3%). Of the 387 SAB cases, 289 (6.5% of the total bacteremia episodes) were included in this study (Fig. 1). Based on the exclusion criteria, 98 cases of SAB were excluded due to 1) patients aged < 18 years (N = 12, 3.1%), 2) hospitalized for at least 4 days after diagnosis of SAB (N = 41, 10.6%), 3) died before starting antibiotics administration (N = 4, 1.0%), 4) discharged or transferred from other facilities within 3 days (N = 6, 1.6%), 5) lost to follow-up at 14 days after diagnosis of SAB (N = 1, 0.3%), 6) clinical diagnosis as contamination (N = 28, 7.2%), and 7) SAB episodes with an interval of less than 3 months in same patients (N = 6, 1.6%). The hospital sizes were categorized as follows; three with > 600 beds, two with 401–600 beds, five with 201–400 beds, and four with ≤ 200 beds. Five hospitals (35.7%) had full-time infectious disease specialists available for consultation. The background and number of SAB episodes at each facility are summarized in Supplementary Table 1.

The clinical characteristics of the patients with SAB are shown in Table 2. The median age of the patients was 77 years (IQR, 62–92 years), and 65.7% were male. Community-onset cases accounted for 51.6% of the cases, and 98 (33.9%) were caused by MRSA. Complicated SAB cases accounted for 85.5% of all episodes, comprising confirmed (152 cases, 52.6%) and potentially complicated SAB cases (95 cases, 32.9%). Nearly one-fourth of the patients (28.4%) did not have any underlying comorbidities, while the proportions of patients with diabetes mellitus (27.7%), malignancy (26.3%), use of immunosuppressive agents (19.7%), and heart failure (19.0%) were high. The total number of patients with multiple comorbidities (≥ 2) was 109 out of 289 (37.7%). The number of cases (proportions) in each of the four groups was 61 (21.1%) for the lowest QIs, 79 (27.3%) for the lower QIs, 96 (33.2%) for the higher QIs, and 53 (18.3%) for the highest QIs. The proportions of sex, site of disease onset, and MRSA across the SAB-QI groups did not differ significantly. The rates of complicated SAB in the lowest (100%) and lower QIs (92.4%) were higher than those in the higher (82.3%) and highest QIs (64.2%). Significant differences were observed in the complicated SAB and uncomplicated SAB categories in the following two-group comparisons: lowest vs. higher QIs, lowest vs. highest QIs, and lower vs. highest QIs. No significant differences were found in the proportions of patients with multiple comorbidities across the QI groups.

**Table 2:** Clinical backgrounds of patients with SAB, stratified by SAB-QI score

		Number (%) of SAB episodes by SAB-QI score group					*p-value*
		Lowest QIs	Lower QIs	Higher QIs	Highest QIs		
		0–6	7–8	9–10	11–13		
Number of cases (total)	289	61 (21.1)	79 (27.3)	96 (33.2)	53 (18.3)		
Age, years (median [IQR])	77 (62–92)	79 (63–95)	79 (64–94)	77 (63–91)	74 (58–90)		0.11
Sex, male	190 (65.7)	41 (67.2)	50 (63.3)	63 (65.6)	36 (67.9)		0.94
Onset, community-onset	149 (51.6)	30 (49.2)	41 (51.9)	47 (49.0)	31 (58.5)		0.70
MRSA	98 (33.9)	22 (36.1)	27 (34.2)	35 (36.5)	14 (26.4)		0.66
SAB category							
Complicated SAB	247 (85.5)	61 (100)	73 (92.4)	79 (82.3)	34 (64.2)		< 0.01*
- Confirmed complicated SAB	152 (52.6)	6 (9.8)	40 (50.6)	72 (75.0)	34 (64.2)		–
- Potentially complicated SAB**	95 (32.9)	55 (90.2)	33 (41.8)	7 (7.3)	0 (0)		–
Uncomplicated SAB	42 (14.5)	0 (0)	6 (7.6)	17 (17.7)	19 (35.8)		< 0.01*
Underlying clinical conditions							
No comorbidity	82 (28.4)	15 (24.6)	23 (29.1)	25 (26.0)	19 (35.8)		0.54
Multiple comorbidities (≥ 2)	109 (37.7)	25 (41.0)	33 (41.8)	37 (38.5)	14 (26.4)		0.62
Diabetes mellitus	80 (27.7)	10 (16.4)	21 (26.6)	34 (35.4)	15 (28.3)		0.08
Malignancy	76 (26.3)	21 (34.4)	20 (25.3)	25 (26.0)	10 (18.9)		0.31
Immunosuppressive agents	57 (19.7)	15 (24.6)	15 (19.0)	18 (18.8)	9 (17.0)		0.74
Heart failure	55 (19.0)	10 (16.4)	17 (21.5)	19 (19.8)	9 (17.0)		0.86
End-stage renal disease***	45 (15.6)	15 (24.6)	10 (12.7)	16 (16.7)	4 (7.5)		0.07
Hemodialysis	25 (8.7)	8 (13.1)	4 (5.1)	10 (10.4)	3 (5.7)		0.29
Liver cirrhosis	19 (6.6)	4 (6.6)	6 (7.6)	5 (5.2)	4 (7.5)		0.92
COPD	16 (5.5)	3 (4.9)	6 (7.6)	6 (6.3)	1 (1.9)		0.55

*SAB* *Staphylococcus aureus* bacteremia. *QI* Quality Indicator. *IQR* interquartile range. *MRSA* methicillin-resistant *S. aureu*s. *COPD* chronic obstructive pulmonary disease

Community onset; identified in outpatients or ≤ 72 h after admission. Nosocomial onset; identified > 72 h after admission

The lowest with 0–6 QIs, lower with 7–8 QIs, higher with 9–10 QIs, and highest with 11–13 QIs

*Significant differences are observed at the category of complicated SAB (confirmed complicated SAB and potentially complicated SAB) and uncomplicated SAB (*p* < 0.01) in the following two-group comparisons; lowest QIs vs higher QIs, lowest QIs vs highest QIs, and lower QIs vs highest QIs

**Potentially complicated SAB was defined as cases without follow-up blood culture

***End-stage renal disease was defined as eGFR < 30 mL/min/1.73m^2^, including patients undergoing hemodialysis

Detailed data on the primary infectious foci and the presence of disseminated lesions in eligible patients are summarized in Supplementary Table 2. Primary bacteremia, in which the infectious foci were unclear, was the most common (87 cases, 30.1%), followed by catheter-related bloodstream infections (CRBSI) (68 cases, 23.5%). The proportion of patients with primary bacteremia tended to increase in the lower-QI groups (47.5% in the lowest, 32.9% in the lower, 27.1% in the higher, and 11.3% in the highest). Disseminated lesions were observed in 99 (34.3%) patients. Bone and joint infections such as osteomyelitis and arthritis accounted for half of these cases (49 cases, 49.5%). Skin and soft tissue infections, including abscesses, were detected in 27 patients (27.3%), and central nervous system infections were diagnosed in 12 patients (12.1%). The proportion of secondary foci was higher in the higher QI group (13.1, 34.2, 38.5, and 50.9%, respectively).

### Distribution of SAB-QI score

**Fig. 2:**
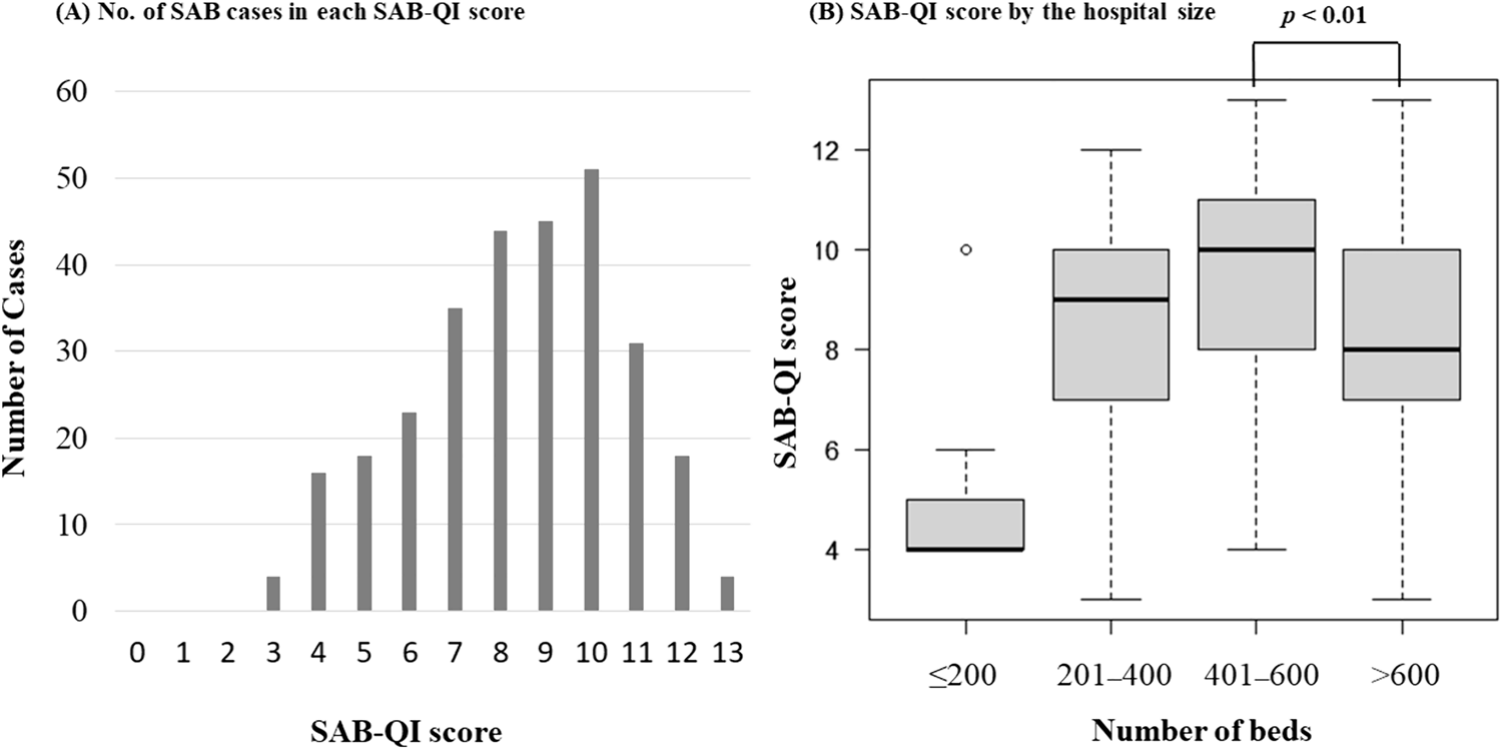
Distribution of SAB-QI score for **A** all SAB cases and **B** by the hospital size. *SAB* *Staphylococcus aureus* bacteremia. *QI* Quality Indicator. *IQR* interquartile range. **A** The median [IQR] SAB-QI score is 9 [6.7–11.3]. **B** The median SAB-QI scores [IQR] in each hospital size were 4 [2.4–5.6] in ≤ 200 beds, 9 [6.9–11.1] in 201–400 beds, 10 [8.0–12.0] in 401–600 beds, and 8 [5.8–10.2] in > 600 beds

We investigated adherence to 13 QIs in the management of SAB. The SAB-QI score was distributed with a range of 3–13 points, with a median of 9 points (IQR: 6.7–11.3) (Fig. 2A). The distribution of the SAB-QI scores was also evaluated according to hospital size (Fig. 2B). A significant increase in the median SAB-QI score was observed in proportion to hospital size in hospitals with ≤ 600 beds (≤ 200 beds vs. 201–400 beds, *p* < 0.01; 201–400 beds vs. 401–600 beds,* p* = 0.016). However, a significant decrease was identified among hospitals with > 600 beds compared to those with 401–600 beds (*p* < 0.01) (Fig. 2B).

### Adherence to SAB-QIs and patient outcome

The overall 30-day mortality rate was 18.0% and the in-hospital mortality rate was 24.9%. Adherence to the SAB-QIs in all, survival, and fatal cases is shown in Table 3. The overall adherence rates were 67.1% for blood culture, 21.1% for echocardiography, 42.6% for source control, 23.2% for antibiotic treatment, and 17.0% for other management. Adherences to four QI categories, including (1) blood cultures (81.9% vs. 34.6%), (2) echocardiography (25.7% vs. 9.6%), (3) source control (51.9% vs. 25.0%), and (4) antibiotic treatment (28.3% vs. 3.8%), was significantly higher in patients who survived. Adherence to 5) other management strategies was also better in the survival cases (20.7% vs. 9.6%), although the difference was not statistically significant.

**Table 3:** The adherence to SAB-QIs and by patient outcome

	Number (%) of episodes			*p-value*
	Total	Survived cases	Fatal cases	
Number of cases	289	237 (82.0%)	52 (18.0%)	
QI category (full, 13 points)	4 (1.4)	4 (1.7)	0 (0)	1.0
1) Blood cultures (maximum, 2 points)	194 (67.1)	176 (81.9)	18 (34.6)	< 0.01*
QI 1–1. Follow-up blood cultures	211 (73.0)	181 (76.4)	30 (57.7)	< 0.01*
QI 1–2. Confirmation of negative blood culture	194 (67.1)	176 (74.3)	18 (34.6)	< 0.01*
2) Echocardiography (maximum, 2 points)	61 (21.1)	56 (25.7)	5 (9.6)	0.025*
QI 2–1. Transthoracic echocardiography**	200 (69.2)	174 (73.4)	26 (50.0)	< 0.01*
QI 2–2. Transesophageal echocardiography***	62 (21.5)	57 (24.1)	5 (9.6)	0.024*
3) Source control (maximum, 1 point**)**	123 (42.6)	110 (51.9)	13 (25.0)	< 0.01*
4) Antibiotic treatment (maximum, 6 points)	67 (23.2)	65 (28.3)	2 (3.8)	< 0.01*
QI 4–1. Initial intravenous antibiotic therapy	287 (99.3)	235 (99.2)	52 (100)	1.0
QI 4–2. Appropriate antibiotic	196 (67.8)	163 (68.8)	33 (63.5)	0.51
QI 4–3. Antibiotic therapy within 24 h	282 (97.6)	232 (97.9)	50 (96.2)	0.61
QI 4–4. Appropriate duration	92 (31.8)	89 (37.6)	3 (5.7)	< 0.01*
QI 4–5. Adjuration according to renal function	259 (89.6)	212 (89.5)	47 (90.4)	1.0
QI 4–6. No intravenous-to-oral switch	288 (99.7)	236 (99.6)	52 (100)	1.0
5) Other management (maximum, 2 points)	49 (17.0)	44 (20.7)	5 (9.6)	0.15
QI 5–1. Infectious disease specialist consultation	69 (23.9)	63 (26.6)	6 (11.5)	0.020*
QI 5–2. Medical discharge summary	133 (46.0)	117 (49.4)	16 (30.8)	0.021*

*SAB* *Staphylococcus aureus* bacteremia. *QI* Quality Indicator

Data given in the parenthesis is the full compliance rate for each QI category

*Significant differences are observed at (1) Blood cultures (QI 1–1,2), (2) Echocardiograph (QI 2–1,2), (3) Source control, (4) Antibiotic treatment (QI 4–4), and QI 5–1,2

**Consisting of 192 cases (66.4%) undergoing transthoracic echocardiography and 8 cases of uncomplicated SAB

***Consisting of 22 cases (7.6%) undergoing transesophageal echocardiography and 40 cases of uncomplicated SAB

The adherence rates for each QI subcategory are listed in Table 3. Significant differences were observed between rates in survival and non-survival cases in the QI 1–1, 1–2, 2–1, 2–2, 3, 4–4, 5–1, and 5–2 subcategories. Specifically, in subcategory QI 1–1, the achievement within 48 h was reported in 105 patients (36.3%). The number of cases of TTE and TEE performed within 5 days was 151 (52.2%) and 9 (3.1%), respectively.

**Fig. 3:**
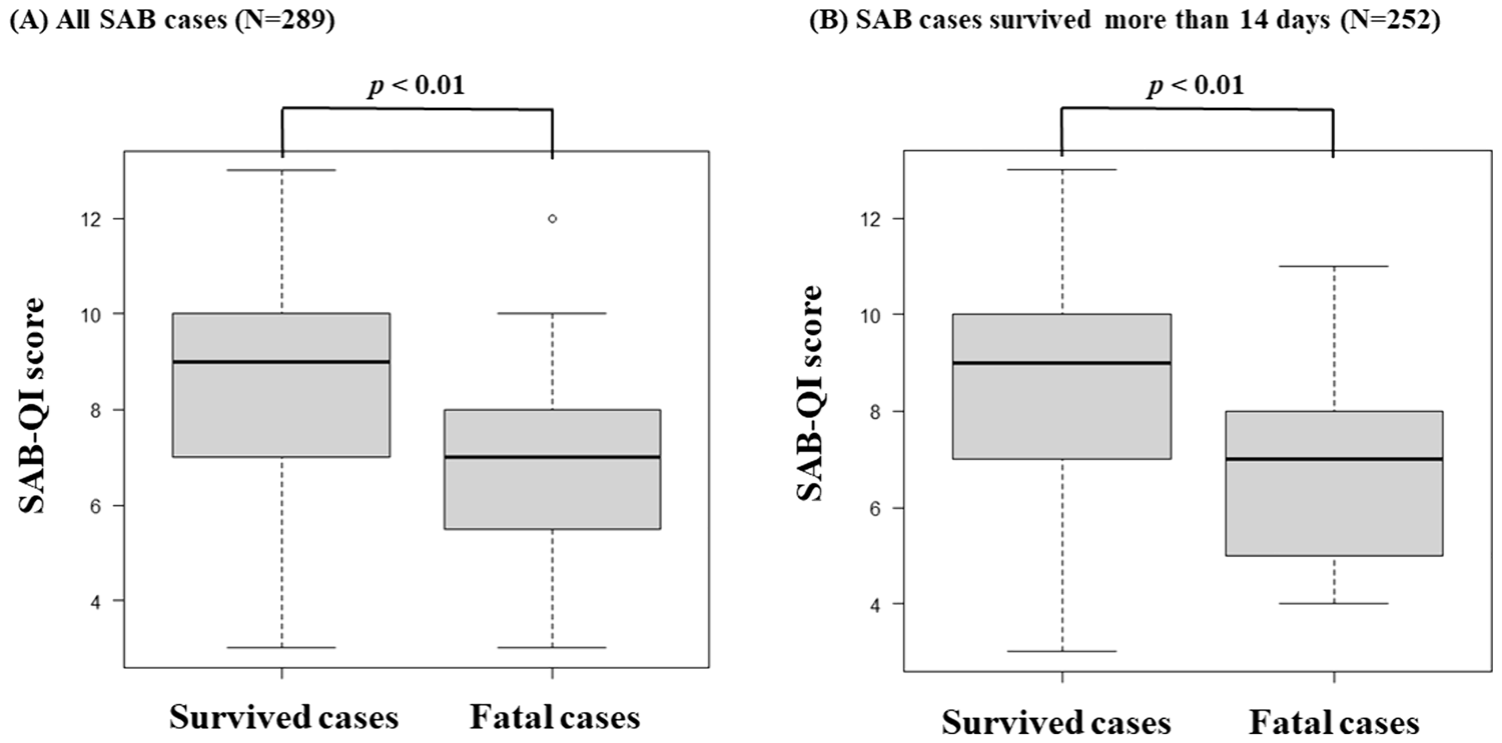
Comparison of SAB-QI scores between survived and fatal cases. *SAB* *Staphylococcus aureus* bacteremia. *QI* Quality Indicator. *IQR* interquartile range. The 30-day mortality rates among **A** all SAB patients and **B** those who survived > 14 days were 18.0% and 6.0%, respectively. **A** The median SAB-QI scores [IQR] (survived vs. fatal cases) were 9 [6.8–11.2] vs. 7 [5.2–8.8] in all SAB cases. **B** The median SAB-QI scores [IQR] (survived vs. fatal cases) are 9 [6.8–11.2] vs. 7 [5.0–9.0] in SAB cases survived more than 14 days

**Fig. 4:**
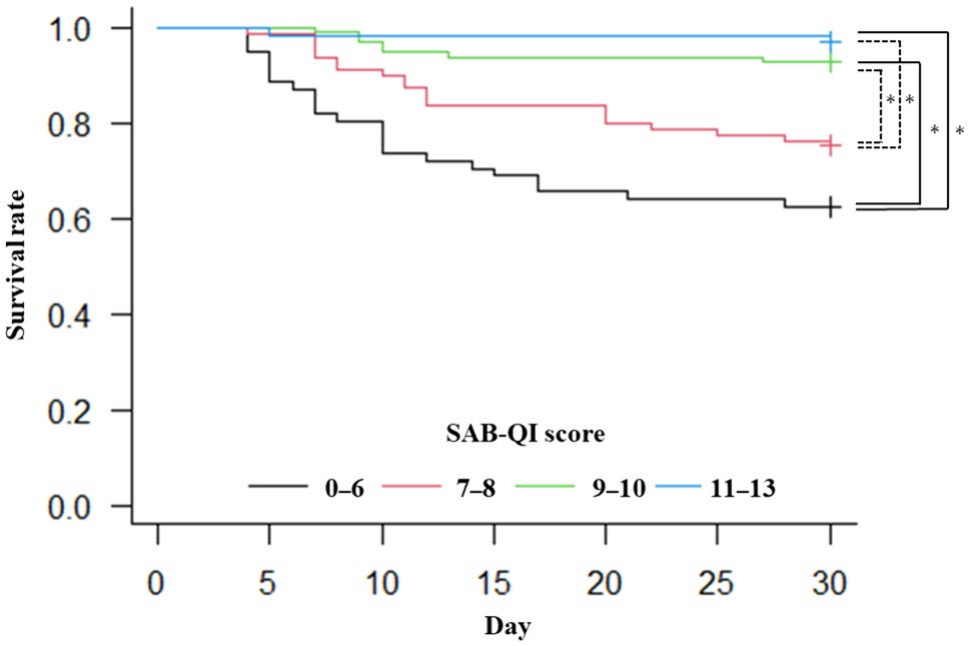
Kaplan–Meier curves for comparing the prognosis of patients with SAB stratified by SAB-QI score groups. *SAB* *Staphylococcus aureus* bacteremia. *QI* Quality Indicator. *Significant differences were observed at the lowest QIs vs. higher and highest QIs (solid lines), and lower QIs vs. higher and highest QIs (dotted lines) (*p* < 0.01). *p-values*: lowest (0–6) vs. lower (7–8) (*p* = 0.073), lowest (0–6) vs. higher (9–10) (*p* < 0.01), lowest (0–6) vs. highest (11–13) (*p* < 0.01), lower (7–8) vs. higher (9–10) (*p* < 0.01), lower (7–8) vs. highest (11–13) (*p* < 0.01), and higher (9–10) vs. highest (11–13) (*p* = 0.39)

The SAB-QI scores were compared between the survived and fatal cases across all SAB cases and among patients who survived beyond two weeks (Fig. 3). In both cases, the SAB-QI scores were significantly higher in the survived patients (median: 9 vs. 7, *p* < 0.01). The 30-day survival rates among the four SAB-QI groups were compared using Kaplan–Meier curves and log-rank tests. The survival rate was significantly lower in the groups with low SAB-QI scores; the lowest and the lower QI groups were significantly different from the highest and the higher QI groups, respectively (*p* < 0.01) (Fig. 4).

### Multivariate analysis to identify prognosticators

The results of the logistic regression analysis are summarized in Table 4**.** The multivariate analysis corroborated that old age (odds ratio [OR], 1.06; 95% confidence interval [CI], 1.02–1.09; *p* = 0.001), methicillin resistance (OR, 3.48; 95% CI, 1.65–7.35;* p* = 0.001), multiple comorbidities (OR, 2.88; 95% CI, 1.41–5.85; *p* = 0.004), and lower SAB-QI score (OR, 0.63; 95% CI, 0.53–0.76; *p* < 0.001) were associated with 30-day mortality in patients with SAB. Site of disease onset (OR, 1.01; 95% CI, 0.50–2.05; *p* = 0.98) and complexity of SAB (OR, 0.44; 95% CI, 0.13–1.50; *p* = 0.19) did not significantly impact patient outcomes.

**Table 4:** Results of logistic regression analysis to identify factors potentially associated with patient prognosis

	Number (%) of episodes			Multivariate analysis		
	Total	Survived cases	Fatal cases	OR	95%CI	*p-value*
Number of cases	289	237	52			
Age, years (median [IQR])	77 (62–92)	76 (61–91)	83 (72–94)	1.06	1.02–1.09	0.001*
Location of onset, community-onset	149 (51.6)	126 (53.2)	23 (44.2)	1.01	0.50–2.05	0.98
Methicillin resistance	98 (33.9)	69 (29.1)	29 (55.8)	3.48	1.65–7.35	0.001*
Complicated SAB	247 (85.5)	200 (84.4)	47 (90.4)	0.44	0.13–1.50	0.19
Multiple comorbidities (≥ 2)	109 (37.7)	80 (33.8)	29 (55.8)	2.88	1.41–5.85	0.004*
SAB-QI score (median [IQR])	9 (6.7–11.3)	9 (6.8–11.2)	7 (5.1–8.9)	0.63	0.53–0.76	< 0.001*

*SAB* *Staphylococcus aureus* bacteremia. *IQR* interquartile range. *OR* odds ratio, *CI* confidence interval. *QI* Quality Indicator

^*^Significant differences are observed in age, methicillin resistance, multiple comorbidities (≥ 2), and SAB-QI score

## Discussion

We highlighted the clinical impact of higher adherence to SAB-QIs on better prognosis in patients with SAB, which can be summarized as follows. First, the higher the SAB-QI score, the better the patient outcome. The 30-day prognosis of patients with higher QI scores (9–10) and the highest QI scores (11–13) was clearly better than that of patients with lower QI scores. Second, the SAB-QI scores varied greatly among the enrolled patients, ranging from 3 to 13, suggesting that the management of patients with SAB differs between institutions and physicians. Adherence to the QIs was worse for echocardiography (21.1%), antibiotic treatment (23.2%), and other management (17.0%). Third, the demographic data obtained in this study will greatly help understand the clinical features of SAB as an intractable disease.

In our patient cohort, SAB accounted for 8.7% of all cases of bacteremia (387/4,448), which may be lower than those previously reported; for example, 16.9% of healthcare-onset and 14.9% of community-onset cases in Canada [24], and 27.1% of nosocomial-onset cases in Japan [25]. MRSA infections were detected in 33.9% of patients in the present study, which is comparable to the findings of previous studies reported in Japan (32.8–44.9%) [9, 14, 26]. As reported previously [27], this is corroborated by data from a Japanese national database indicating MRSA isolation rates of 30.7–67.3% in recent years [28]. Potential infectious foci of SAB were reported to be bone and joint (2.4–24.2%), skin and soft tissue (12.5–17.0%), CRBSI (11.6–12.3%), IE (5.5–10.9%), respiratory tract (5.9–15.3%), surgical site (5.3%), and urinary tract infections (3.9%), and primary/unknown cases (19.1–42.7%) [7, 25, 26]. Of the 289 cases finally included, the common infectious foci were primary bacteremia (30.1%) and CRBSI (23.5%), indicating that CRBSI was relatively common in our patients.

The proportion of complicated SAB was high (85.5%) in the present study compared to previously reported data (305/530, 57.5%) [7]. Over one-third (34.3%) of the SAB cases were accompanied by disseminated lesions, especially those involving osteomyelitis and arthritis (49.5%). These results emphasize that systemic examination and management are indispensable for patients with SAB. The lower the SAB-QI score, the higher the primary bacteremia and fewer the disseminated lesions. This could be attributed to the absence of follow-up blood cultures and inadequate systemic assessment, resulting in an insufficient classification of uncomplicated and complicated cases.

The clinical utility of QI-oriented management in ensuring the quality of medical care for patients with SAB [10, 13–19]. A promising relationship between high adherence to QIs and favorable prognosis has been corroborated worldwide [14, 29, 30]. Our data underscore the clinical significance of adherence to recommended SAB-QIs in improving patient outcomes. In the present study, the median SAB-QI score was 9 points (maximum, 13 points). It is not feasible to compare our data with those of previous studies, and the adequacy of this compliance rate for maintaining medical safety in real-world settings remains unclear. However, SAB-QI scores can be applied to interhospital comparisons or longitudinal evaluations in hospitals.

In our study, follow-up blood cultures after initiating antimicrobial therapy were not performed in one-third of the SAB cases. A recent study reported that follow-up blood cultures were tested in only 18.8% of cases managed in Japanese emergency and critical care departments, indicating inadequate management of patients with SAB [26]. Repeated blood culture testing is indispensable to distinguish between complicated and uncomplicated SAB. Without proper diagnosis, it is impossible to establish an appropriate period of antibiotic treatment [10], possibly increasing the likelihood of therapeutic failure. The fact that most previous studies have included confirmation of negative blood culture as one of the recommended QIs suggests its clinical importance [14–16, 18, 19]. In our study, a criterion for defining uncomplicated SAB was negative blood culture results during follow-up. Potentially complicated SAB cases were classified as either uncomplicated or complicated SAB if follow-up blood culture testing had been performed. In this study, the prevalence rates were 14.5–47.4% for uncomplicated SAB and 52.6–85.5% for complicated SAB, which is equivalent to or surpassed complicated SAB prevalence rates reported previously (46.9%) [13].

Interestingly, our data demonstrated a relationship between hospital size and the SAB-QI score. The median SAB-QI score significantly increased as the hospital volume increased. However, paradoxically, the SAB-QI score of the largest hospital group (> 600 beds) was lower than that of the second-largest hospital group (401–600 beds). Generally, the larger the hospital, the better equipped the testing facilities and the more specialists are employed there. Thus, this result is informative as the clinical management of patients with SAB is not necessarily better in high-volume hospitals. We assume that it is difficult for specialized departments (such as Infectious Diseases [ID], Cardiology, and Cardiac Surgery) and laboratory divisions (such as Microbiology and Echocardiography) to fully collaborate in large hospitals. Studies on hospital volume and the quality of medical care have been conducted in various medical fields. For instance, the results of a retrospective multifacility cohort study indicated that the survival rate of patients with ovarian cancer may increase depending on hospital volume [31]. Admission to a high-volume hospital may be associated with lower mortality or higher quality of medical care, although some data suggest that large hospitals do not necessarily provide better medical care to every patient in proportion to hospital size [32, 33]. The diagnostic accuracy and treatment strategies at small-scale hospitals may be suboptimal mainly due to the unavailability of in-hospital facilities for blood culture and a lack of current medical knowledge. Considering that SAB is also a common disease in such small hospitals, it is imperative to evaluate and monitor the quality of care provided to patients with SAB there as well. To draw solid conclusions, the association between SAB management quality and hospital size should be further explored in future studies.

The mortality rate of SAB is approximately 20–30% in developed countries despite effective antibacterial therapy and source control [2]. Despite the implementation of active antimicrobial stewardship and infectious disease consultation (11–24%) [34] or evidence-based bundle intervention (17–22%) [13], favorable reductions in mortality have yet to be achieved in patients with SAB. The overall 30-day mortality rate in the present study was 18.0%, with 37 patients (71.2%) dying within 14 days after SAB diagnosis. Among the patients who survived for > 14 days, the 30-day mortality rate was 6.0%. Thus, the prognoses in our patient cohort were favorable as reported in a previous Japanese study (3.4–10.0%) [14]. The cause of the disparity in prognoses reported in Japan and those reported in other developed countries remains unclear. Variations in the prevalence of circulating pathogenic strains across countries may be a contributing factor [35]. Given that SAB may precipitate multiple complications that require prolonged treatment (> 30 days), the assessment of long-term prognosis is imperative. However, prognostic evaluation following inter-hospital transfer posed a challenge in this retrospective study. Older age, the presence of one or more comorbidities, and methicillin resistance are reported potential predictors for mortality in patients with SAB [2, 36]. Our study also corroborated that age, methicillin resistance, multiple comorbidities, and lower SAB-QI scores are associated with 30-day mortality in patients with SAB.

The landscape of therapeutic strategies for patients with SAB is evolving. In standard practice, patients with SAB undergo at least 2 weeks of intravenous antibiotic therapy, and the treatment period is extended to 4 to 6 weeks in complicated cases [5, 37]. The optimal duration remains a subject of debate and two non-inferiority RCTs are currently underway; the SAB7 trial aims to evaluate the efficacy of 7- and 14-day antibiotic treatment in low-risk patients [38], and the SAFE trial aims to compare 4- and 6-week intravenous antibiotic therapy in patients with complicated SAB including native valve infective endocarditis [39]. Another RCT has indicated that selected low-risk patients with uncomplicated SAB can be safely and effectively managed with early oral switch therapy [40]. Although increased evidence on a shorter antibiotic strategy is favorable, patients with the poor prognosticators warrant particular attention to improve their prognosis.

Our data clearly demonstrated that the SAB-QI score was significantly lower in fatal cases (median: 7 vs. 9, *p* < 0.01) (Fig. 3). Moreover, the lower the SAB-QI score, the lower the 30-day survival rate (Fig. 4), as previously reported [13, 14, 34]. Additionally, multivariate analysis indicated that lower SAB-QI scores are associated with poor prognoses in patients with SAB. Our SAB-QI was developed from the original 25 QIs [10], but its clinical validity remains unclear. However, based on our data, we suggest that the SAB-QI has the potential to play an important role in improving the prognosis of patients with SAB by ensuring the quality of medical care.

Here, we discuss ways to increase SAB-QI scores and enhance patient management. Similar to QI-oriented management, five core interventions for SAB have recently been proposed in a review article: (1) appropriate anti-staphylococcal therapy, (2) screening echocardiography, (3) assessment of metastatic phenomena and source control, (4) decision on antimicrobial therapy duration, and (5) ID consultation [41]. Consultation with ID physicians is quite difficult in Japan, where specialized doctors are not readily available [42]. Systematic education and training curricula for undergraduate students and young doctors are required to increase the number of ID physicians. Another approach, the development of antimicrobial stewardship teams or programs, and bundle management, has been reported to improve the prognosis of patients with SAB [13, 14, 34, 43]. In the absence of ID physicians, especially in small hospitals, such collaborative work may contribute to an increase in SAB-QI scores, subsequently leading to a better patient prognosis.

Our study has two strengths. First, the SAB-QI score, which was established by modifying the original 25 QIs [10], was used for clinical evaluation. Our score was simplified and thus could be made more easily available in any healthcare setting. Second, 289 cases from 14 hospitals were included; thus, generalizability of the analyzed data is warranted. However, this study had several limitations. First, we retrospectively collected clinical and microbiological data from the medical records. Thus, there may be errors or misunderstandings in the past data. In addition, considering the difficulty of evaluating past data, the timeframes to achieve each QI score were not fully defined. Second, although a description of the clinical course of SAB in the medical discharge summary is included in the SAB-QIs [16], its relationship with prognosis is unclear because the patient may have died before completing the summary. Third, several cases of SAB have been treated with ceftriaxone, particularly in cases complicated by central nervous system infections; however, the clinical validity of ceftriaxone treatment in cases of SAB remains uncertain. An additional QI proposal for SAB complicated with central nervous lesions is expected in the future. Fourth, SAB cases from small-scale hospitals (fewer than 200 beds) represented a minor proportion of the cases (4.5%), potentially leading to a selection bias in the population. Fifth, of the 98 excluded cases, nearly half (42 cases, 42.9%) were reported at a single acute care hospital, which may suggest a selection bias. The primary reasons for exclusion were clinical diagnosis of contamination (18 cases, 42.9%); and early transfer and hospitalization duration less than 4 days after diagnosis (13 cases, 31.0%). Given that *S. aureus* infrequently causes contamination during blood culture testing [44], this observation should be further examined. However, validation of diagnoses via retrospective analysis posed a challenge. In addition, the high patient transfer rate in acute care hospitals is unavoidable. Sixth, the assessment of mortality might have necessitated quantification of the severity of chronic underlying conditions using the Charlson Comorbidity Index. However, because of the retrospective nature of this study, the accurate collection of essential data was not possible. Instead, we compared the frequencies of multiple comorbidities in each QI group. Seventh, the study lacked a clear definition of the appropriate timing for source control measures when collecting the data. This may have caused an overestimation of QI values. Finally, adherence to SAB-QIs may be worse in patients with poor prognoses related to underlying clinical conditions. In such cases, the clinician had potentially adopted a more conservative approach, which may have reduced QI compliance. However, the effects of such confounding factors were not fully evaluated in this retrospective study.

## Conclusion

Our multifacility study suggests that higher adherence to the SAB-QIs is associated with better patient outcomes. Age, MRSA, the presence of two or more underlying chronic diseases, and low SAB-QI score were identified as prognostic factors in patients with SAB. The wide range of SAB-QI scores in the enrolled patients suggests large gaps in the management quality of patients with SAB. Multifaceted educational approaches are required to improve adherence to SAB-QIs.

### Supplementary Information

Below is the link to the electronic supplementary material.

Supplementary file1 (DOCX 25 KB)

## Data Availability

Detailed data are available upon request from the corresponding author.
