# Supplementary material for: Adherence to and clinical utility of “quality indicators” for Staphylococcus aureus bacteremia: a retrospective, multicenter study
Source: Infection. 2024 May 10;52(4):1527–38. doi: 10.1007/s15010-024-02284-z (PMC11289132; doi:10.1007/s15010-024-02284-z)
Supplement: Supplementary file 1 — Supplementary file1 (DOCX 25 KB) [file 15010_2024_2284_MOESM1_ESM.docx]

**Supplementary Table 1. Backgrounds and the numbers of SAB cases in participating hospitals.**

| **Participated hospitals** | **Number of beds** | **Full-time infectious disease specialist available for consultation** | **All SAB cases** | **Enrolled SAB cases** | **Excluded cases** | **SAB-QI score (Median [IQR])** |
| --- | --- | --- | --- | --- | --- | --- |
| Kurashiki Central Hospital | 1172 | No | 117 | 75 | 42 | 8 [6.1–9.9] |
| Okayama University Hospital | 855 | Yes | 40 | 31 | 9 | 8 [5.4–10.6] |
| Okayama Medical Center | 609 | Yes | 45 | 36 | 9 | 10 [7.7–12.3] |
| Tsuyama Chuo Hospital | 515 | Yes | 56 | 46 | 10 | 9 [7.2–10.8] |
| Okayama City Hospital | 400 | Yes | 50 | 38 | 12 | 10 [8.1–11.9] |
| Okayama Kyoritsu General Hospital | 318 | Yes | 33 | 25 | 8 | 9 [7.5–10.5] |
| Marugame Medical Center | 300 | No | 7 | 7 | 0 | 9 [6.0–12.0] |
| Kurashiki Medical Center/Clinic | 269 | No | 6 | 5 | 1 | 5 [3.0–7.0] |
| Okayama Kyokuto Hospital | 214 | No | 5 | 2 | 3 | 9 [8.5–9.5] |
| Brain Attack Center Ota Memorial Hospital | 213 | No | 13 | 11 | 2 | 8 [6.1–9.9] |
| Tamano City Hospital | 199 | No | 2 | 2 | 0 | 5 [4.5–5.5] |
| Niimi Central Hospital | 115 | No | 1 | 1 | 0 | 4 [4.0–4.0] |
| Kasaoka City Hospital | 99 | No | 3 | 3 | 0 | 6 [3.6–8.4] |
| Mabi Memorial Hospital | 80 | No | 9 | 7 | 2 | 4 [3.5–4.5] |
|  |  | Total | 387 | 289 | 98 | 9 [6.7–11.3] |

SAB: *Staphylococcus aureus* bacteremia. QI: Quality Indicator. IQR: interquartile range.

**Supplementary Table 2. Primary infectious foci and the presence of disseminated lesions of patients with SAB.**

|  |  | **Number (%) of SAB episodes** | | | |
| --- | --- | --- | --- | --- | --- |
|  |  | **Lowest QIs** | **Lower QIs** | **Higher QIs** | **Highest QIs** |
|  | **SAB-QI score** | **0**–**6** | **7**–**8** | **9**–**10** | **11**–**13** |
| Number of cases (Total) | 289 | 61 | 79 | 96 | 53 |
| **Primary infectious foci** |  |  |  |  |  |
| Catheter-related bloodstream infection | 68 (23.5) | 10 (16.4) | 19 (24.1) | 27 (28.1) | 12 (22.6) |
| Osteomyelitis, arthritis | 35 (12.1) | 4 (6.6) | 11 (13.9) | 8 (8.3) | 12 (22.6) |
| Skin and soft tissue infection, abscesses | 26 (9.0) | 5 (8.2) | 8 (10.1) | 9 (9.4) | 4 (7.5) |
| Urinary tract infection | 20 (6.9) | 6 (9.8) | 6 (7.6) | 4 (4.2) | 4 (7.5) |
| Infective endocarditis | 16 (5.5) | 0 (0) | 2 (2.5) | 4 (4.2) | 10 (18.9) |
| Respiratory tract infection | 16 (5.5) | 4 (6.6) | 3 (3.8) | 9 (9.4) | 0 (0.0) |
| Surgical site infection | 10 (3.5) | 1 (1.6) | 0 (0) | 6 (6.3) | 3 (5.7) |
| Graft infection, endovascular infection | 4 (1.4) | 1 (1.6) | 1 (1.3) | 2 (2.1) | 0 (0) |
| Others | 7 (2.4) | 1 (1.6) | 3 (3.8) | 1 (1.0) | 2 (3.8) |
| **Primary bacteremia** | 87 (30.1) | 29 (47.5) | 26 (32.9) | 26 (27.1) | 6 (11.3) |
| **Disseminated lesions** |  |  |  |  |  |
| No | 190 (65.7) | 53 (86.9) | 52 (65.8) | 59 (61.5) | 26 (49.1) |
| Yes | 99 (34.3) | 8 (13.1) | 27 (34.2) | 37 (38.5) | 27 (50.9) |
| Osteomyelitis, arthritis | 49 (49.5) | 5 (62.5) | 14 (51.9) | 12 (32.4) | 18 (66.7) |
| Skin and soft tissue infection, abscesses | 27 (27.3) | 3 (37.5) | 5 (18.5) | 11 (29.7) | 8 (29.6) |
| Central nerve system infection | 12 (12.1) | 0 (0) | 1 (3.7) | 5 (13.5) | 6 (22.2) |
| Graft infection, endovascular infection | 6 (6.1) | 0 (0) | 1 (3.7) | 4 (10.4) | 1 (3.7) |
| Thrombophlebitis | 5 (5.1) | 1 (12.5) | 1 (3.7) | 3 (8.1) | 0 (0) |
| Others | 13 (13.1) | 1 (12.5) | 4 (14.8) | 5 (13.5) | 3 (11.1) |

SAB: *Staphylococcus aureus* bacteremia. QI: Quality Indicator. IQR: interquartile range.

The primary infectious foci are determined by the medical record review. Others in infectious foci contain sinusitis, cholecystitis, enteritis, thrombophlebitis, central nervous system infection. Others in disseminated lesions contain pneumonia, empyema, prostatic abscess, peritonitis, urinary tract infection, pleuritis.
